# Preliminary investigations for the development of a virtual reality-based English-language communication program: Using the Delphi method

**DOI:** 10.1371/journal.pone.0264850

**Published:** 2022-03-15

**Authors:** Jihyo Kim, Seong Man Park, Meeran Joo, Jongtae Park, Young-Lim Lee, Jong-Hwa Jang, Walcir Cardoso

**Affiliations:** 1 Department of Career Education, College of Liberal Arts, Cheonan, Chungcheongnam-do, Republic of Korea; 2 Department of General English Education, College of Liberal Arts, Cheonan, Chungcheongnam-do, Republic of Korea; 3 Department of Predentistry, College of Dentistry, Cheonan, Chungcheongnam-do, Republic of Korea; 4 Department of Psychology and Psychotherapy, College of Health and Welfare, Cheonan, Chungcheongnam-do, Republic of Korea; 5 Department of Dental Hygiene, College of Health and Welfare, Cheonan, Chungcheongnam-do, Republic of Korea; 6 Department of Education, Faculty of Education, Montreal, Quebec, Canada; Dai Hoc Duy Tan, VIET NAM

## Abstract

The purpose of this study was to gather opinions from experts via the Delphi method to inform the future development of a virtual reality based English language communication program for university level students in Korea. The participants, who consisted of a panel of experts and professors who majored in English language and multimedia education, completed three Delphi surveys based on Context, Input, Process, and Product evaluation, which is referred to as CIPP. In the first Delphi survey, the participants answered multiple choice questions and open-ended questions related to four areas relevant to the development of a virtual reality based program. Based on their answers, a second Delphi survey was designed to determine the participants’ level of agreement with the appropriateness of the questions related to the four areas. In the third Delphi survey, participants were shown the results (mean, standard deviation, median, interquartile range, consensus chart, and convergence degree) and were asked to confirm or modify their answers based on the other participants’ answers. According to the analysis of the Delphi survey results, need for the development of a virtual reality based English language communication program was suggested, and recommendations were made regarding the content and application of the program.

## Introduction

The ability to communicate in English has increasingly become recognized as an essential skill due to globalization. This is particularly relevant when considering the growing importance of university-level English-language education programs that can train students for this globalized world in which the ability to communicate in English is required in most fields.

To prepare students appropriately, Korean universities have attempted to implement more practical English as a Foreign Language (EFL) programs by focusing on communication skills rather than traditional teaching methods (e.g., reading comprehension, reading classic novels) in order to increase the students’ ability to communicate in English. In fact, in the last twenty years, the goal of the university-level EFL program has shifted to focus on strengthening the four English skills (reading, writing, listening, speaking) and on developing communication skills [1] in a realistic and meaningful learning environment.

Information and communication technology offer effective tools to create the desired meaningful learning environment. The widespread use of smart devices (e.g., smartphones, tablets, e-books, smart TVs) has contributed to technological tools becoming widely adopted in pedagogical contexts [2]. This has facilitated the implementation of a variety of computer-assisted language learning (CALL) tools in the classroom. In fact, recently, Virtual Reality (VR) as well as Artificial Intelligence (AI) have been introduced in pedagogical contexts [3–10]. An increasing number of research on these novel technologies have examined cases where VR or AI software were designed and employed to enhance English communication skills [4–11]. These studies have highlighted the benefits of the realistic and meaningful online reality that can be created for a learner to practice communicating in English at any time on their own. Within the EFL context, in particular, Lan [8] points out such three benefits of the use of virtual contexts through the action research in Taiwan as limitless provision of students’ English learning opportunities regardless of time and space, provision of fun and meaningful EFL learning activities for students in 3D virtual environments, and enhancement of students’ EFL performances. In a similar vein, Swier and Peterson [6] claim the usefulness of 3D digital games and virtual space for language learners within the Japanese EFL context at a higher education level through their qualitative research with language teachers in Japan. They maintain that the development of technology on the provision of virtual space for English learning and mobile devices can provide language learners with mixed reality, where the real world is combined with virtual reality especially in the EFL context where there are limited chances for the learners to be exposed to the use of the target language in a real life situation.

Although English-language programs enhanced with VR technology provide academic benefits [7, 11–13], the educational content available on the existing programs have limitations. First, it is difficult to find a program where learners can customize the educational content to focus on and to adapt the difficulty level according to their own needs (e.g., educational background, cultural background, and experience in preparation for exam, preferred topics) [14]. Research has shown that there is a high demand for the development of a wider variety of content topics learners can interact with using VR programs [15]. It has also been shown that English and Korean professors recognize the importance of the possibility for the learner to customize the level of difficulty of the content in the program [15]. Through the analysis of 29 VR related research articles during the period from 2004 to 2013, Lin and Lan [12] also confirmed the need for the development of VR programs considering learner differences including interactive communication, cultural awareness, affections, behaviors, beliefs, intelligence, and language proficiency. Therefore, there is a need for existing VR-based English-language educational programs to become more learner based.

Second, most research on these programs has taken place in elementary schools and has focused on the development of reading, writing, and vocabulary, as well as its effect on affective factors [16, 17] but not on the development of communication skills. Lin and Lan [12] also point out that the efficacy of different VR educational contexts focusing on learner interactive communication should be examined further in future research. Therefore, there is a need for research to examine the effects of VR-based English-language educational programs on both the development of communication skills as well as at the university level in the Korean educational context.

Third, sufficient practical suggestions have yet to be provided on the application of virtual environments especially in educational settings at a school level regardless of the numerously claimed benefits of virtual environments in educational settings [13]. This, Kim, Lee, and Thomas [13] claim that diversity, verification, and specification should be considered for the application of virtual worlds for educational purposes in order to provide practical ways to use virtual environments in the field of education.

Finally, the existing research regarding the development of a software that combines educational content and new technology are mostly experimental, and thus, it is necessary to reflect on and expand the experts’ opinions in the early stages of development in order to reach educational effectiveness. To address this gap in research, this research study aimed to conduct a series of surveys via the Delphi method, allowing researchers to analyze the opinions of an expert group by collecting, combining, and quantifying the experts’ opinions on multiple occasions [17].

Accordingly, there is a need for research into the development of a VR-based English-language communication program at a university level. Thus, the purpose of this study was to gather the opinions of experts to develop such a program. The purpose of this study was two-fold. The first purpose was to collect the experts’ opinions regarding the development of a VR-based English-language communication program using the Delphi method. The second purpose was to propose a plan to develop a VR-based program for university-level students in Korea based on the Delphi results.

## Background

### English-language education in Korean universities

Korean universities have recognized the importance of English-language education programs and have made English a required course for graduation to improve the students’ level of English [18, 19]. In fact, after the 1990s, there was a shift in English-language education at the university level from a focus on teaching grammar and literature to implementing the communicative approach. This has led to the integration of a variety of new teaching and learning approaches over the last few decades. Since then, research has examined the shift in focus of these English-language university courses in Korea to shed light on the effectiveness of the shift as well as to learn about the students and their teachers’ needs [7, 20]. Research has also been conducted to identify the most beneficial approaches to teaching English at the university level [3]. This research has motivated universities to modify their English courses. In fact, universities evolved to focus on strengthening the four English skills (reading, writing, listening, speaking) and designing intensive courses to develop communication skills [1]. For example, universities have integrated TOEIC (Test of English for International Communication) and TOEFL (Test of English as a Foreign Language) classes and English for Specific Purposes (ESP) courses to focus on the development of business English skills [21]. Universities have also increased the number of teachers who are native English speakers and have developed practical English courses in terms of culture and everyday life where students can learn English while discovering a variety of English-speaking cultures [22].

Not only have teaching approaches evolved since the 1990s, but teaching tools have also evolved as a result of new technology. Research has been conducted to examine the potential of these new technological tools in pedagogical contexts by particularly with a focus on the potential of computers and smart devices (e.g., smartphones, tablets, e-books, smart TVs). More recently, the development of VR, AI, and augmented reality (AR) software has led to an increase of research conducted to examine the educational potential of these software.

University-level English-language education has become increasingly interested in teaching methods that foster a communicative approach based on the educational theory of constructivism [23]. Constructivism refers to a process where the learner shapes an intellectual model in a self-directed way [24]. According to constructivism, learners are active subjects while professors are cognitive facilitators who provide directions and models for assigned tasks [25]. Many classrooms have adopted this educational theory by implementing CALL tools in their English-language courses (e.g., computers and mobile phones, online learning materials). For example, English-language programs implementing CALL tools (e.g., 3D printing, VR, AR, and AI) have become widely adopted as they foster the development of the learners’ communication skills. Moreover, the appearance of these CALL tools have led to innovative teaching approaches to take over and replace traditional teaching methods and become the center of curriculum design [13]. In order to successfully implement these new teaching methods, it is necessary to effectively use digital environments or digital-based learning content during classes. Thus, it would be desirable to suggest appropriate directions for research on the educational and practical applications of virtual environments in educational settings.

However, there is a lack of CALL tools that offer content that can be selected and adapted by professors according to the learning objectives and level of the students. Therefore, this research will investigate the need to develop a VR-based English-language communication program offering customizable content according to learners’ own needs for a variety of teaching environments.

### Virtual Reality (VR)

Virtual Reality (VR) is a technology that allows users to experience a virtually-simulated world that is similar to reality as it involves visual, auditory, and tactile senses, as well as to communicate with information from that world via a computer (desktop/hard VR) or a VR headset (3D VR) [26]. The principle of VR, which is an integrated technology involving science, technology, computer graphics, interaction technology between human and computer, and artificial intelligence, is to create a virtual three dimensional environment through a computer in which users can experience sensory simulations in real time and have a sense of reality through the interaction between the users and the computer [9]. In addition, VR is a technology that utilizes virtual contents through immersive equipment, which can create scenarios and reproduce real environments [27].

Seminal research on VR consisted of pilot studies examining the new technology and its applicability in a variety of fields (e.g., media, medicine, education) [28–30]. In the field of education, VR technology has been used to host educational content as the technology has the potential to provide a more vivid learning experience [27]. Additional research has been conducted to investigate how this novel CALL tool may affect learners in a variety of areas (e.g., interest in the English course, achievement levels, level of immersion in the learning experience, satisfaction of English-language education) [31, 32].

### VR-based English-language communication programs

Previous research has examined the integration of VR in English-language pedagogical contexts in Korea and around the world to determine the benefits of the technology and its potential in the classroom. Chung [10] investigated the effects of a VR platform in an English-language course with first-year university students. In this study, the active learning approach implemented via the use of the VR platform fostered the shift from a teacher-centered learning environment to a student-centered environment as the technology offered the learner to possibility to navigate the VR software anytime and anywhere. Yeh, Tseng, and Heng [33] also investigated the way of enhancing students’ intracultural learning through VR among EFL students within the Taiwan context and claimed that students were able to gain better intracultural awareness and improved their English vocabulary skills through the interactive features of VR technology. In a similar vein, Saito [34] revealed that the use of VR in English education for university students in Japan increased students’ engagement, reduced anxiety in their English language learning, and promoted their active learning.

Bonner and Reinders [35] examined a variety of language classes to investigate the effects of AR and VR functions. They investigated English as a Second Language courses where learning tasks were completed via CALL tools (e.g., using smartphones, AR software, and VR platforms) and pointed out potential limitations of the technology in the classroom. With regard to the effectiveness of VR and AR technologies on language learning, Huang, Zou, Cheng, and Xie [36] claimed that those technologies could help students promote learning, improve motivation, reduce learning anxiety, and have positive attitudes towards using those technologies in their language learning in their systematic review of current studies on VR and AR tools on language learning. VR programs have also been integrated in communication courses when combined with a computer-mediated communication (CMC) tool allowing the learner to communicate with the software [28]. In fact, Kim [28] integrated a 3D VR-based program combined with a CMC tool to enhance communication skills. This research suggests that the learning experience was enhanced by the use of all senses as opposed to the traditional teacher-centered approach.

The use of a CMC tool in a VR program was also examined in a context where learners of English had the opportunity to have a conversation with virtual characters whose voices were provided by native speakers of English [7]. The research shows that the program offers an environment to improve communication skills as a built-in system provided feedback on mistakes in pronunciation. In fact, the learners’ pronunciation was assessed by giving a score on the learners’ voices.

Research on VR has also examined the effect of the technology on affective aspects [4, 37]. In fact, Jin [4] studied the effects of an online extracurricular 3D VR game on the participants’ affective intercultural ability. TraceEffect was the educational game used for the study. It is a game that provides a virtual world where the users can visit many US cities and experience different cultures. In the game, players complete missions by communicating and interacting with English-speaking characters. It was found that the attributes that are related to intercultural ability (e.g., attitude, knowledge, interpretation, making association, criticizing a culture) were all developed by the participants who played TraceEffect. The results suggest that the VR game was effective in enhancing their intercultural ability as it provided the participants with an opportunity to learn new cultures. However, there is a need to consider the optimal learning environment, evaluate the pros and cons of new learning methods, and understand the learning experience from the development phase of the program since no complimentary study was done to investigate usage patterns (e.g., the amount of time each player spent exploring new cultures through the game, the number of times each player participated in the program). In a similar vein, Zheng, Young, Brewer, and Wagner [37] also investigated the effect of a 3D game-like virtual environment on affective factors and self-efficacy for Chinese EFL learners in a middle school-based setting in China. They investigated the effects of an online game called Quest Atlantis, which allows learners to use English in their self-selected activities in virtual environments, on the learners’ attitudes toward English learning and self-efficacy in their EFL learning. The results of their investigation confirmed the Chinese EFL learners’ positive attitude toward English and the enhancement of their self-efficacy in English since the virtual English learning environment provided less stressful and more natural learning contexts. Both studies [4, 37] confirm the positive influence of the use of virtual environments in English education settings, in particular, on affective aspects. Furthermore, their research on VR suggests that the effects of virtual environments for English learning would be more meaningful especially within the EFL context (e.g., Korean & China) where learners have limited opportunities to use English in natural settings outside of the classroom. Thus, in EFL contexts, the virtual educational environments can provide learners with diverse chances to experience the actual use of English in real-life contexts with native English speaking people and with stress-free and enjoyable English learning contexts, which eventually leads to learners’ long-term engagement [37] in their English learning.

VR-based English-language communication programs have also been developed to include AI algorithms and voice recognition. In fact, Cheon [11] developed one using Google Card Board VR and Google Speech API. In this program, the voice-recognition tool separates the sentence said by the user into words that are entered in the database. An answer is then provided to the user based on the highest probability to create a conversation as fluid as possible between the user and the virtual character. Cheon [11] determined the adequacy of the program by comparing the English voice-recognition entries from the users with the ratio of appropriate answers from the virtual character.

As seen, research on the implementation of VR-based English-language communication programs has been conducted to identify the potential of VR programs on the improvement of English communication skills. However, research on the use of VR in pedagogical contexts is still in its preliminary stage as most are exploratory studies that examine possible applicability or that investigate the students’ perspectives [35]. In a similar vein, Parmaxi [38] also points out that a sound pedagogical grounding along with technical configuration of VR should be further investigated regardless of the great potential of the VR technology as a valuable tool in the field of language learning. In this regard, this research attempts to do a study on developing an effective VR-based English-language educational program by gathering opinions from experts, breaking away from previous studies that have focused on the effects of applying the new technology.

## Methods

### Research design

#### Participants

The participants in this study consisted of a group of experts on English-language and multimedia education from Korea (OOU 2019-11-014-002). This study was approved by the OO UNIVERSITY INSTITUTIONAL REVIEW BOARD. Fourteen teachers and professors who majored in English-language education or multimedia education, and who teach English-language education at an elementary, secondary, or university level, were selected. The main purpose of this study is to verify the content validity of VR-based English communication content, and for this purpose, English education and multimedia education experts as content experts were recruited as the target population of this study. Based on Anderson’s [39] suggestion that a small group of 10 to 15 experts could obtain useful results, a total of 15 experts were selected. The results were presented based on a panel of 14 experts who responded to the Delphi survey conducted three times in total.

All the participants agreed to participate in the research and written informed consent was obtained. The list of participants with their respective participation rates are shown in Table 1.

**Table 1:** List of participants.

Description of participants (*n* = 14)				Participation rates		
No.	Years of experience	Current teaching position	Highest degree obtained	1^st^ Survey	2^nd^ Survey	3^rd^ Survey
P1	6	University profession	Ph.D.	Yes	No	No
P2	9	University professor	Ph.D.	Yes	Yes	Yes
P3	15	University professor	Ph.D.	Yes	Yes	Yes
P4	12	Primary-school teacher	Master’s	Yes	Yes	Yes
P5	7	University professor	Ph.D.	Yes	Yes	Yes
P6	24	Secondary-school teacher	Ph.D.	Yes	Yes	Yes
P7	24	University professor	Master’s	Yes	Yes	Yes
P8	14	University professor	Ph.D.	Yes	Yes	Yes
P9	14	University professor	Ph.D.	Yes	Yes	Yes
P10	10	University professor	Ph.D.	Yes	Yes	Yes
P11	15	University professor	Ph.D.	Yes	Yes	Yes
P12	25	University professor	Ph.D.	Yes	Yes	Yes
P13	14	University professor	Ph.D.	Yes	Yes	Yes
P14	15	University professor	Ph.D.	Yes	Yes	Yes

#### Materials

*The Delphi surveys*. As part of the Delphi method, Delphi surveys were created. The Delphi survey is a group facilitation technique, which is an iterative multistage process, designed to transform opinion into group consensus [40]. The Delphi technique is a useful decision-making tool for reaching consensus among experts in a certain field through surveys that enables a group to efficiently respond to complex problems at the overall level rather than the individual level [40]. This Delphi technique is a kind of research method designed for the purpose of collecting the opinions of relevant experts for policy making or business planning in order to obtain consensus through expert opinion surveys several times on content that is not yet known or has not reached a certain consensus. Thus, the Delphi survey method was employed in this study, since it is considered to be a useful methodology for collecting the opinions of experts to design a VR-based communication program and verifying the content validity of the provisionally designed program.

Evaluation categories were selected. As well, the evaluation scale (open item, Likert scale 1–5) was developed based on responses obtained from surveys administered to a panel consisting of teachers and an expert group. The teachers were experts in English-language education and multimedia education. The expert group mostly consisted of professors with expertise in English-language education at the university level and with experience in developing multimedia educational programs as they are familiar with the needs of the target end-users of the program. The development of the Delphi surveys began with the extraction of the four areas and associated content and was followed by the development of the evaluation scale.

#### Revision process to verify and finalize tool

An expert group comprised of professors in the education department (three educational technology specialists, one educational evaluation specialist) revised the Delphi surveys to ensure the validity of the tool’s content. Some of the recommendations taken into consideration were the following: adding multimedia data and program prototype; increasing the total number of questions to ensure the reliability of responses; adding consideration of importance for decision making on development decision; subdividing learning objectives into understanding, application, and application to real situations; and providing instructions on how to use VR program.

#### Procedure

The Delphi method was used with the participants to obtain information related to existing VR-based English-language communication programs. In this study, a questionnaire tool with CIPP model was developed, and Delphi survey was conducted three times in total in order to collect basic data of VR-based English communication program development.

In the first Delphi survey, experts’ opinions on four areas consisting of multiple-choice questions and open-ended questions were collected by mixing unstructured response forms and structured questionnaires. The main purpose of the first survey was to examine the divergent perception of experts, and then collect opinions from them. The first Delphi survey, which took place over a period of four weeks, consisted of both multiple-choice questions (MCQ) and open-ended questions (OEQ) to obtain the experts’ opinions regarding four areas: (1) the program’s necessity and academic diagnosis, (2) the education content, (3) the teaching method and evaluation, and (4) the results, applications of the four English skills, and other issues to consider for program design. To verify the validity of the questionnaire, two English education experts and one expert in Education participated to review the validity of the questionnaire. The content of the questions in the first survey, the number of questions, and the format of the questions are shown in Table 2.

**Table 2:** First Delphi survey with the application of CIPP model (2019.11.04 ~ 2019.12.06).

Area	Criteria	Number of questions	Format	
			MCQ	OEQ
1. Program’s necessity and academic diagnosis (Situation assessment)	• Problems in existing speaking and listening based programs	3	Yes	Yes
	• Necessity and educational expected effect	3	Yes	Yes
2. Education content (Input assessment)	• Learning objectives	2	No	Yes
	Academic content and components	2	No	Yes
	• VR learning situations and content	2	No	Yes
3. Teaching method and evaluation (Process assessment)	• Learning content and method	2	Yes	Yes
	• Level of conversation	3	Yes	Yes
	• Duration of learning	2	Yes	Yes
	• Evaluation method and evaluation factor	3	Yes	Yes
4. Results and applications of four English skills (Output assessment)	• Results application (universities, professors, students)	3	Yes	Yes
	• Other issues: Teaching content for enhancement in communication skills and other opinions	1	No	Yes

The second Delphi survey took place over a period of seven weeks. 93 questions were created based on the responses from the first Delphi survey regarding the four areas in order for the experts to determine their level of agreement with the appropriateness of the questions. The responses were on a 5-point Likert Scale ranging from 1 (very inappropriate) to 5 (very appropriate). The median, mean, and interquartile range were computed based on the responses from the multiple-choice type questions from the first survey. The content of the questions in the second survey, the number of questions, and the format of the questions are shown in Table 3.

**Table 3:** Content of the second and third Delphi surveys with the application of CIPP model (2nd: 2019.12.16 ~2020.02.02/ 3rd: 2020.02.16 ~ 2020.02.26).

Area	Criteria	Category	Number of questions
1. Program necessity and academic diagnosis (Situation assessment)	• Problems in existing programs	• Appropriateness of existing speaking-based course content	2
		• Appropriateness of existing listening-based course content	
	• Necessity of program	• Necessity for an English conversation program	6
		• Necessity for development of VR-based English-language education content	
		• Necessity for pronunciation education content including accent, tone, and speed	
		• Necessity for development of content including frequency-based expressions by native English speakers	
	• Educational effect	• Effect of conversation practice through VR (conversation skills, academic motivation, global competence)	8
2. Education content (Input assessment)	• Academic goal	• Method of developing English communication skills	7
		• Experience communicating in English within a real life-like environment	
		• Development of learning language and English-language abilities through the process of learning cultures	
		• Increase in confidence, and decrease of negative sentiment toward English	
	• Course content	• Learning pronunciation and communication skills through VR animation, listening and speaking evaluation in vocabulary and sentence structure	7
	• VR content and learning situations	• Validity of different situations in VR (Campus, Trip, Culture, etc.)	16
3. Teaching method and evaluation (Process assessment)	• Course content and method	• Curriculum, use of extra-curricular activities, learning through learner-focused educational contents	6
	• Conversation content, difficulty, and duration	• Factors that impact difference in difficulty	30
		• Different conversation content based on difficulty	
		• Listening practice, speaking practice	
	• Evaluation method and factors to consider	• Evaluation of appropriate conversation skills in situations	8
		• Matching degree between a native speaker’s speech and learner’s speech	
		• Detailed feedback on the learner’s errors and mistakes	
4. Results and applications (Output assessment)	• Applications of results by university, professor, student	• Application in teaching and learning, classes, and extra-curricular activities	3
		• Global competence, learner-tailored learning	

The third Delphi survey took place over a period of ten days. The participants were shown the results of the second Delphi survey, so that the respondents could compare their opinions with those of the other experts. The results consisted of the expert’s own responses, as well as the mean, standard deviation median, and quadrant range for the consensus items, which are statistical analysis results for the second questionnaire so that respondents could refer to the opinions of other experts in re-evaluating the importance of each item. After consulting the results, the participants were prompted to either agree or disagree with the opinions of other experts and to provide an explanation when their responses deviated from those of the others. In this study, as a result of the 3rd survey, most of the items showed a high level of consent and agreement between the panels, so the panel question was terminated with the 3rd survey.

The summarized Delphi survey procedures and analysis stages are shown in S1 Fig.

#### Research tools and analysis method

*Tool development*. Evaluation categories were selected, and evaluation scale was developed through surveys done by teachers who are experts in English language education and multimedia education, as well as by an expert council. An expert group was mostly consisted of professors who currently have experience in English language education at universities and also have experience in developing multimedia educational programs because it is considered that the possible users of developing program in this study are university students, and the purpose of this study was to enhance availability of English language education and learning at a university-level. VR-based English communication development was done through the following procedures: Extraction of program development areas and criteria -> Selection of items by criteria -> Scale development.

*Verification and development of tool through revision (final version)*. The developed tool went through the process of revision and correction of the content validity by professors in the field of education (i.e., 3 educational technology specialists & 1 educational evaluation specialist), and then the final Delphi survey tool was developed. The revised contents in the draft survey tool based on a consensus as follows: ‘Additional offering of multimedia data, and program prototype’, ‘Adjustment of the total number of questions in order to increase the reliability of responses’, ‘Additional consideration of importance for decision making on development decision’, ‘Subdivision of learning objectives into understanding, application, and application to real situations’, ‘Instructions on how to use VR program’.

#### Data analysis

Quantitative data from the first Delphi survey consisted of the responses to the MCQs which were converted into numeric value and entered in the SPSS statistical program (25.0). Descriptive statistics (mean, standard deviation, median, interquartile range) were computed. Qualitative data from the first Delphi survey consisted of the responses to the OEQs.

In terms of the second and third Delphi surveys, the quantitative data consisted of the responses to the questions on a 5-point Likert Scale ranging from 1 (very inappropriate) to 5 (very appropriate) related to the level of agreement with the appropriateness of the questions. The descriptive statistics were also computed for the second and third Delphi surveys. In addition, consensus chart and convergent degree were computed to draw out the sum of the opinions of participants (see below for Formulas 1 and 2) (Lee, 2001). According to previous studies on the conditions for consensus chart and convergent degree, the members have come to an agreement when the consensus is above 0.75 and when the convergent degree is below 0.50 (Lee, 2008).


$$Consensus=1‐\frac{\left(Q3-Q1\right)}{Mdn}$$ (Eq 1)

$$Convergent\;degree=\frac{\left(Q3-Q1\right)}{2}$$ (Eq 2)

## Results

### First Delphi survey results

The information in Table 4 highlights some of the answers collected from the MCQs and OEQs from the first Delphi survey. The answers have been organized within the four areas.

**Table 4:** Overview of responses from MCQs and OEQs from first Delphi survey.

Area	Criteria	Responses
1. Necessity of VR-based programs and academic diagnosis	• Difficulties in English communication	• Fear of making mistakes, confidence in pronunciation, lack of sentence structure
		• Lack of motive to enhance English communication skills
		• Lack of exposure to conversing with English speakers
		• Difficulties in applying English-language education to actual English communication situation
		• Absence of learning conversation strategies needed to converse with English speakers
	• Necessity	• Necessity for conversation practice for real-life situations
		• Necessity for English-language education through conversations between human and computers
		• It is expected that learning English communication through movie scenes will be effective
2. Academic content of VR-based English-language communication program	• Constituents	• Additional explanation on program manipulation and content
		• Creation of various content persistent of English learning
		• Development of program where learners have the option of choosing their goals and appropriate contents
	• Academic content	• Diversity in listening speed of native conversation
		• Provide indirect experience on different cultures of English-speaking countries
		• Composing of the program to allow English learning through the process of learning the culture
		• English conversation scenes: shopping, work out, beauty, entertainment, K-pop, health, accidents, job interview…
3. Learning method and evaluation of VR-based program	• Evaluation	• Rather than suggesting in %, provide learners with practice questions to do additional training or offer corrections on sentence errors
		• Provide learners with congruence of accent curves while practicing on English conversation.
4. Necessity of VR-based program and academic diagnosis	• Difficulty	• In need of division of difficulties in conversation
		• Adjustment of difficulty based on grammar, vocabulary, sentence length, amount of conversation, conversation contents, pronunciation speed, sentence structure (complex sentence, inversion), situation (everyday life, education)
		• Academic word list (intermediate level), tongue twister (advanced level)
5. Results of VR-based program and application plan	• Usages in teaching method	• Make use of it in flipped classrooms
		• Create a for teachers to use as course materials
		• Use in extra-curricular activities or intensive courses
		• Establish a learning situation where a debate or a conversation with several people at the same time is possible

### Second and third Delphi survey results

The results of the second and third Delphi surveys consist of the participants’ responses to the 5-point Likert Scale questions regarding their level of agreement with the appropriateness of the questions. Most respondents maintained their original answers and when they did make changes, they followed to the consensus of the majority. The responses have been divided into the four areas as follows:

#### Area 1: Necessity for a program and academic diagnosis

The results of the participants’ level of agreement with the appropriateness of the questions related to the first area, Necessity for a Program and Academic Diagnosis, for the second and the third Delphi surveys are shown in Table 5 and S2–S4 Figs. In terms of the first criterion, *problems in existing programs*, by the end of the third Delphi survey, the participants had a low level of agreement with the appropriateness of the content and method for both existing speaking programs (M = 2.69) and listening programs (M = 2.69).

**Table 5:** Level of agreement with the appropriateness of the questions (second and third Delphi survey results).

Categories for each criterion	Mean/5		SD		Mdn/5		Consensus		Convergence	
	2^nd^	3^rd^	2^nd^	3^rd^	2^nd^	3^rd^	2^nd^	3^rd^	2^nd^	3^rd^
Problems in existing programs										
Speaking course contents	2.69	2.69	.63	.63	3	3	.67	.67	.50	.50
Listening course contents	2.92	2.69	.76	.75	3	3	.50	.67	.75	.50
Necessity of program										
English conversation program	4.77	4.77	.44	.44	5	5	.90	.90	.25	.25
VR-applied content	4.77	4.69	.44	.48	5	5	.90	.80	.25	.50
Pronunciation content	4.54	4.38	.52	.51	5	4	.80	.75	.50	.50
Content from native speakers	4.77	4.85	.83	.55	5	5	1.00	1.00	.00	.00
Expected educational effect										
Effect of multimedia learning	4.31	4.31	.63	.63	4	4	.75	.75	.50	.50
Competence enhancement	4.46	4.38	.52	.51	4	4	.75	.75	.50	.50
Effect of VR situations	4.31	4.31	.75	.63	4	4	.75	.75	.50	.50

Regarding the second criterion, *necessity of an enhanced program*, there was a high level of agreement with the necessity to develop an English conversation program by the third Delphi survey (M = 4.77), which meant that recognition for the necessity was high. The need for content from native speakers obtained the highest level of agreement by the third Delphi survey (M = 4.85).

When looking at the third criterion, *expected educational effect*, by the third Delphi survey, there was a high level of agreement for effects from multimedia learning (M = 4.31) and learning in VR situations (M = 4.31), and many responded that this program is expected to be effective in improving English-language abilities.

Excluding the criterion of *problems of current programs*, there was a consensus among experts by the third Delphi survey on the *necessity of a program* and *expected educational effects* criteria.

#### Area 2: Education content of the program

The results of the participants’ level of agreement with the appropriateness of the questions related to the second area, Education Content of the Program, for the second and the third Delphi surveys are shown in Table 6 and S5–S10 Figs. In terms of the first criterion, *academic goal*, the categories that obtained the highest level of agreement with the appropriateness of the content by the third Delphi survey included the goal to have authentic interaction (M = 4.15), the goal for improvement of competence (M = 4.31), and the goal for an increase in the interaction confidence level (M = 4.62). These results show that the respondents considered the development of English-language abilities which include learning about and understanding English-speaking cultures, as well as learning a language in a VR space, being an important objective.

**Table 6:** Level of agreement with the appropriateness of the questions (second and third Delphi survey results).

Categories for each criterion	Mean/5		SD		Mdn/5		Consensus		Convergence	
	2^nd^	3^rd^	2^nd^	3^rd^	2^nd^	3^rd^	2^nd^	3^rd^	2^nd^	3^rd^
Academic goal										
Choice of learning method	3.69	3.62	.74	.65	4	4	.75	.75	.50	.50
Need for improvement	3.92	3.85	.64	.55	4	4	.88	.88	.25	.25
Understanding how to improve	4.08	4.08	.49	.49	4	4	1.00	1.00	.00	.00
Authentic interaction	4.23	4.15	.73	.69	4	4	.75	.75	.50	.50
Improvement of competence	4.31	4.31	.63	.63	4	4	.75	.75	.50	.50
Interaction confidence level	4.46	4.62	.66	.65	5	5	.80	.80	.50	.50
Course content										
Learning with VR	4.58	4.54	.52	.52	5	5	.80	.80	.50	.50
Listening/Speaking evaluation	4.42	4.46	.52	.52	4	4	.75	.75	.50	.50
Practice with VR	4.33	4.31	.49	.48	4	4	.75	.75	.50	.50
VR learning situations										
Library										
Borrow or return a book	4.50	4.46	.52	.52	5	4	.78	.75	.50	.50
Request a book	4.58	4.46	.52	.66	5	5	.80	.80	.50	.50
Use library facilities	4.33	4.31	.65	.63	4	4	.75	.75	.50	.50
Café										
Order coffee	4.92	4.85	.29	.38	5	5	1.00	1.00	.00	.00
Use café facilities	4.67	4.62	.49	.51	5	5	.80	.80	.50	.50
Complain and refund	4.83	4.77	.39	.44	5	5	1.00	.90	.00	.25
Tourist spot										
Ask for direction					5			.80	.38	.50
Inquire/Purchase a product	4.75	4.69	.45	.48	5	5	.85	.80	.50	.50
Exchange/Refund a product	4.58	4.54	.67	.66	5	5	.80	.80	.38	.50
Concert	4.75	4.69	.45	.48		5	.85			
Request for program							5	.75	.50	.50
Purchase ticket/seat	4.33	4.31	.78	.75	5	4	.78	.90	.00	.25
Critique performances	4.83	4.77	.39	.44	5	5	1.00	.80	.50	.50
	4.58	4.62	.67	.51		5	.80			

In terms of the second criterion, *course content*, the mean of the level of agreement with the appropriateness of the content for each category was above 4. Moreover, the participants reached an agreement on all categories of the criterion as the consensus level was above .75 and the convergence degree was below .50. On the second Delphi survey, the participants identified their level of agreement with the appropriateness of following topics in VR learning situations enumerated from highest to lowest level of agreement: travelling, interview in English, movies or English drama, public institutions, emergency situations, university campus, K-culture (e.g., Korean pop & food culture), sports, English-speaking cultures, cooking, shopping, beauty, fashion, time travel to the past or future, medical knowledge, Do-it-Yourself projects, reading.

In terms of the third criterion, *VR learning situations*, the participants rated their level of agreement with the appropriateness of the conversation topics considering the difficulties (advanced, intermediate, beginner) of the VR situations. There was a high level of agreement by the third Delphi survey in the situations related to the library: borrowing or returning a book (M = 4.46), requesting a book (M = 4.46), using library facilities (M = 4.31). In terms of situations related to tourist spots, there was a high level of agreement for the topic of asking for direction (M = 4.69), inquiring or purchasing a product (M = 4.54), and exchanging or refunding a product (M = 4.69). As seen in the responses of the experts on the learning objectives of the program that ‘it is important to learn a language while indirectly experiencing English-speaking cultures’, there is a high necessity in developing a virtual reality situation where it is hard to experience. There was a high level of agreement in the two remaining categories of topics related to a café (M > 4.50) and to a concert (M > 4).

#### Area 3: Learning methods and assessment of the program

The results of the participants’ level of agreement with the appropriateness of the questions related to the third area, Learning Methods and Assessment of the Program, for the second and the third Delphi surveys are shown in Table 7 and S11 and S12 Figs. In terms of the first criterion, *difficulty level factors*, the participants rated their level of agreement with the appropriateness of the difficulty levels that were suggested by a group of experts who recommended that the program should be developed by subdividing into difficulties depending on the learner’s ability to communicate in English. The level of agreement with the appropriateness by the third Delphi survey was high regarding the topic or situation (M = 4.54) and sentence length (M = 3.23).

**Table 7:** Level of agreement with the appropriateness of the questions (second and third Delphi survey results).

Categories for each criterion	Mean/5		SD		Mdn/5		Consensus		Convergence	
	2^nd^	3^rd^	2^nd^	3^rd^	2^nd^	3^rd^	2^nd^	3^rd^	2^nd^	3^rd^
Difficulty-level factors										
Level of learner test	2.18	2.23	.87	.83	2	2	.50	.50	.50	.50
Vocabulary	2.92	2.77	1.08	1.01	3	3	.42	.67	.88	.50
Sentence length	3.42	3.23	1.31	1.09	4	3	.29	.50	.05	.75
Pronunciation speed	1.45	1.69	.52	.75	1	2	.00	.50	.50	.50
Sentence structure	2.73	2.85	.91	1.07	3	3	.67	.50	.50	.75
Topic/Situations	4.62	4.54	.51	.52	5	5	.80*	.80	.50	.50
Providing feedback										
Dialogue	3.92	4.00	.64	.41	4	4	1.00	1.00	.00	.00
Pronunciation/fluency	4.46	4.38	.52	.51	4	4	.75	.75	.50	.50
Corrective feedback	4.69	4.62	.48	.51	5	5	.80	.80	.50	.50

In terms of the second criterion, *providing feedback*, the category related to corrective feedback obtained the highest level of agreement of the appropriateness by the participants (M = 4.62). However, the category related to feedback during dialogue obtained the lowest level of agreement (M = 4.00). The results imply that it is necessary to provide them feedbacks that help them enhance communication abilities more practically rather than simply providing feedbacks with graphs showing accuracy on their communication ability.

#### Area 4: Program results and applications

The results of the participants’ level of agreement with the appropriateness of the questions related to the fourth area, Program Results and Applications, for the second and the third Delphi surveys are shown in Table 8 and S13 Fig. The results show that by the third Delphi survey, there was a higher level of agreement with the appropriateness of a program in extra-curricular courses or intensive courses (M = 4.38) than in a university teaching and learning context (M = 4.00).

**Table 8:** Level of agreement with the appropriateness of the questions (second and third Delphi survey results).

Categories for each criterion	Mean/5		SD		Mdn/5		Consensus		Convergence	
	2^nd^	3^rd^	2^nd^	3^rd^	2^nd^	3^rd^	2^nd^	3^rd^	2^nd^	3^rd^
Applications of the program										
University teaching and learning	4.08	4.00	.64	.58	4	4	.88	1.00	.25	.00
In class (flipped learning, test)	4.23	4.31	.73	.48	4	4	.75	.75	.50	.50
Extra-curricular/Intensive course	4.46	4.38	.52	.51	4	4	.75	.75	.50	.50
Preview, review, assignments	4.38	4.31	.65	.63	4	4	.75	.75	.50	.50

## Discussion

The purpose of this study was to lay the groundwork for a university-level VR-based English-language communication program. Using the Delphi method with experts of English-language and multimedia education, a preliminary investigation was conducted to examine existing English-language communication programs to inform the development of an enhanced VR-based program. The overall findings and implications of this study in terms of the four areas, which are 1) necessity for a program and academic diagnosis, 2) education content of the program, 3) learning methods and assessment of the program, 4) program results and applications, derived from the Delphi surveys are as follows.

In terms of the first area, Necessity for a Program and Academic Diagnosis, the findings of the Delphi surveys indicate that the experts had a low level of agreement regarding the appropriateness of the existing speaking and listening programs. However, the level of agreement related to the necessity of an enhanced program was high, specifically when the program contained content from native speakers and VR-applied content. Moreover, according to the first Delphi survey, it was shown that students lacked experience in using conversation strategies needed for conversations with English speakers, and that learning from lectures did not necessarily transfer to real-life situations. These results suggest the need for the development of an English-language communication program where students can practice conversing with English speakers in authentic and realistic situations that simulate real life. The results of this study corroborate the findings of Joh [1] and Chang [22] in which they suggested that English native-speaking professors and communicatively-oriented English courses are needed. In addition, in line with the results of this study, Lan [8] also claims that authentic VR contexts have the significant beneficial effects on learners’ communication abilities in the EFL context.

In terms of the second area, Education Content of the Program, the academic goal criterion indicates that experts had a relatively high level of agreement regarding the appropriateness of the goal to have authentic interaction, the goal for improvement of competence, and the goal for an increase in the interaction confidence level. These results suggest that through an enhanced program, it is suggested that learners could overcome the fear of communicating with English speakers, increase their confidence when using English and increase their overall ability to communicate in English while learning about the cultures of English-speaking countries. Learning about and understanding other cultures is particularly valuable in the context of globalization. VR can effectively simulate situations that would not otherwise be available for the learners due to time, location, and monetary constraints. Therefore, VR can provide an enhanced educational experience that develops intercultural abilities through experiencing and interacting with people from different cultural backgrounds. Within the same area, the results of the Delphi surveys related to the course content criterion revealed that there was a high level of agreement with the appropriateness of content that allows for the learning of English with VR, the evaluation of speaking and listening, and the practicing of communication with VR. Furthermore, the responses from the experts indicate that the most appropriate VR simulation topics consists of travelling. Other VR simulation topics were also considered to be appropriate, such as interviews in English, movies or English dramas, public institutions, and emergency situations. The topics chosen for the third criterion, VR learning situations, consisting of library, café, tourist spots, and concerts along with their difficulty levels (beginner, intermediate, advanced) received a high level of agreement of their appropriateness from the expert group. These VR situations are similar to those used in the VR-based program examined by Jin [4] in which users could visit different cities of the US, encounter US culture and complete game tasks by communicating in English. In both Jin [4] and this study, learning situations deemed appropriate for a VR simulation are ones that cannot easily be experienced or encountered by the learners. Therefore, a VR-based English-language communication program may positively impact the learners’ interest in the English course, achievement levels, level of immersion in the learning experience, satisfaction of English-language education [8, 31, 32, 37]. However, more research should be conducted to establish data base and develop a variety of content that can be selected by learners or teachers to personalize the learning experience.

In terms of the third area, Learning Methods and Assessment of the Program, Delphi surveys examined the factors that should be considered in terms of feedback (the level of difficulty, the type of assessment, and the method). In terms of the first criterion, *difficulty-level factors*, the level of agreement of the appropriateness revealed to be high for topics or situations, sentence length, and sentence structure. When considering the second criterion, *providing feedback*, the level of agreement for the method was high for corrective feedback and low for pronunciation or fluency correction. These results corroborate those of Kim et al. [7] where they established a system that provides feedback on the errors found in a learners’ conversation and scored the users’ voice to give feedback on pronunciation. Moreover, according to the results of the first Delphi survey, there is a need for an integration of an evaluation component in the program. Therefore, it could be suggested that an evaluation component could improve the learners’ communicative skills by providing them with feedback on their speech and identifying the specific areas with which learners struggle the most.

In the last area, Program Results and Applications, the Delphi surveys revealed a higher level of agreement of the appropriateness of a program in extra-curricular activities and intensive courses than in university teaching and learning situations. While Chung [10] introduced VR-based English-language programs in university courses, more research should be conducted to identify other pedagogical contexts in which VR-based programs could be integrated (e.g., flipped learning, blended learning).

## Conclusion

This study is meaningful in that it laid the foundation for developing a program that can improve English communication skills by applying VR technology to college students.

As a result of SWOT analysis of the Delphi survey results to develop a VR-based English communication program in Table 9, three factors were derived, each of strengths, weaknesses, opportunities, and threats, and based on these, four response strategies were suggested. First, it is necessary to develop a communication program that applies VR technology as the SO strategies, and it is necessary to compose contents with high frequency of use by native speakers in an environment similar to reality. In addition, a strategy for nurturing global competence by developing contents that allow students to experience various cultural and language learning at the same time was presented. Second, as the WO strategies, the guidelines for the continuity and linkage of learning using VR contents and the contents with the difficulty level in consideration of each situation and topic of conversation were developed. A strategy was established to develop contents that provide specific feedback on sentence errors. Third, as the ST strategies, a strategy to develop self-directed English learning contents so that learners can practice communication on their own initiative and to prepare various contents to selectively use contents according to the needs of learners and instructors was established. In addition, a strategy to develop contents that consider the high frequency of English communication in real life such as tourist spot, library, café, and concert. Fourth, as the WT strategies, it is necessary to develop various class types such as flipped learning and blended learning to utilize VR teaching media, and it is also necessary to educate and train on teaching and learning strategies required for conducting classes using VR-based contents in actual classroom settings. To prepare an educational environment for VR-based classes, a support strategy such as budgeting was established.

**Table 9:** SWOT analysis matrix.

**Internal factors** **External factors**	**Strengths**	**Weaknesses**
	S1 Prompting interest and motivation for learning English	W1 Continuity of learning using VR contents
		W2 Difficulty in creating contents per level considering the difficulty level
	S2 English communication experience in a realistic environment is possible S3 Global competency cultivation	
		W3 Technical difficulties in evaluating and providing feedback on learning
**Opportunities**	**SO(Strategy)**	**WO (Strategy)**
O1 Discontent with the existing listening and speaking oriented communication improvement program	SO1 Development of a communication program using VR technology	W1. Development of guidelines for continuity and connection of learning using VR contents
	SO2 Composition of contents with high frequency of use by native speakers in an environment similar to reality	
		WO2 Development of contents with the difficulty level considering the topic of conversation by situation
O2 Demand for contents that native speakers use frequently	SO3 Development of contents that can experience various cultural acquisition and language learning at the same time	WO3 Development of contents that provide specific feedback on sentence errors
O3 Demand for English communication experience in VR		
**Threats**	**ST(Strategy)**	**WT(Strategy)**
	ST1 Develop a program that enables learners to practice communication on their own	
T1 Necessity of developing various types of classes to utilize the program		WT1 Development of various types of classes (flipped learning, blended learning) which use VR teaching media
		WT2 Education for the development of teaching competency using VR contents
T2 Necessity of instructor training for classes using VR based teaching media	ST2 Develop a program that enables learners and instructors to selectively use various contents	
		WT3 Budgeting for preparing an educational environment for VR-based classes
	ST3 Develop a program in consideration of the high frequency of communication (tourist spot, library, café, concert)	
T3 Establishment of an environment for classes using VR based teaching media		

There are some limitations in this study that should be considered for future research. Indeed, the participants who participated in this study were university professors only from Seoul and Chungcheongnam-do, and there were only 14 participants. Additionally, they were experts in English-language education, and multimedia education, but they did not have experience in the development of VR-based programs. In this study, English education and multimedia education experts were selected as an expert group to verify the content validity of the VR-based English communication program. In selecting the panel, representativeness, appropriateness, professional knowledge ability, sincerity of participation, and the number of participants were taken into consideration, but it has a limitation in that it has not been able to include a wider group of experts with experience in VR-based education. Therefore, in the future, it would be required to compare and analyze results by organizing a group of experts in consideration of whether they have experience in performing VR-based classes and whether they have experience in developing VR-based educational contents. Therefore, the results of this study should be generalized with caution. Based on these limitations, further research should be conducted to survey more experts in different geographical areas and with more expertise on VR.

Furthermore, future research should also be conducted to collect empirical data on VR-based English-language communication programs. For the purpose of this study, a group of experts was surveyed to gather their opinions related to VR-based English-language communication programs with the purpose of improving the quality of those programs and ultimately developing VR-based programs according to the recommendations. However, in the future, in order to verify the validity of the program design and interface, research should be conducted by forming a group of experts including educational engineering experts and VR program developers.

Finally, this study revealed that it would be beneficial for VR-based programs to include content that could be customized by learners and professors to personalize the learning experience. Therefore, in the future, research should be conducted to examine learners’ needs for VR-based English communication programs targeting learners as well as instructors. In this regard, research that reflects the opinions of various expert groups and users in the program development by comparatively analyzing the needs of VR developers, instructors, and learners. In addition, more research should be conducted to collect and analyze quantitative and qualitative data to identify the specific needs of university students and professors.

## Supporting information

S1 Fig Delphi survey and analysis stage. (TIFF)

S2 Fig Problems in existing programs. (TIFF)

S3 Fig Necessity of program. (TIFF)

S4 Fig Expected educational effect. (TIFF)

S5 Fig Academic goal. (TIFF)

S6 Fig Course content. (TIFF)

S7 Fig VR learning situations: Library. (TIFF)

S8 Fig VR learning situations: Café. (TIFF)

S9 Fig VR learning situations: Concert. (TIFF)

S10 Fig Difficulty-level factors. (TIFF)

S11 Fig (TIFF)

S12 Fig Providing feedback. (TIFF)

S13 Fig Applications of the program. (TIFF)
