# Supplementary figures and images for: Preliminary investigations for the development of a virtual reality-based English-language communication program: Using the Delphi method
Source: PLoS One. 2022 Mar 15;17(3):e0264850. doi: 10.1371/journal.pone.0264850 (PMC8923470; doi:10.1371/journal.pone.0264850)

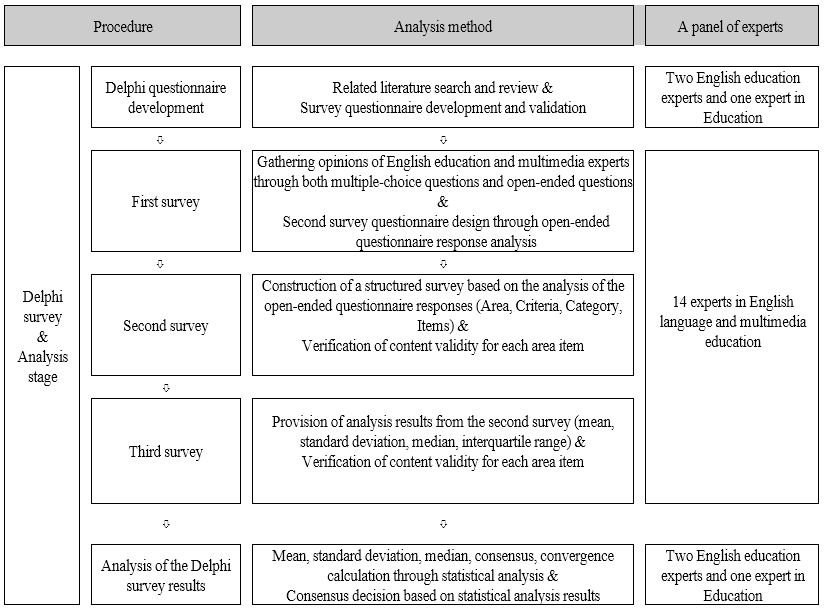

Supplement: S1 Fig — (TIFF) [file pone.0264850.s001.tiff]

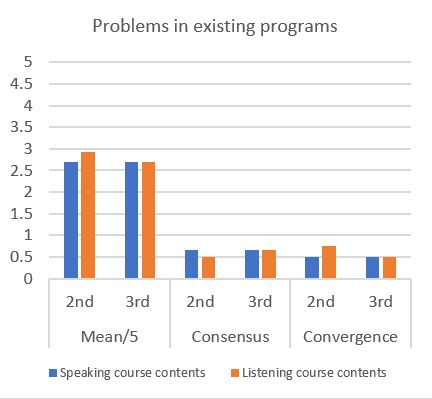

Supplement: S2 Fig — (TIFF) [file pone.0264850.s002.tiff]

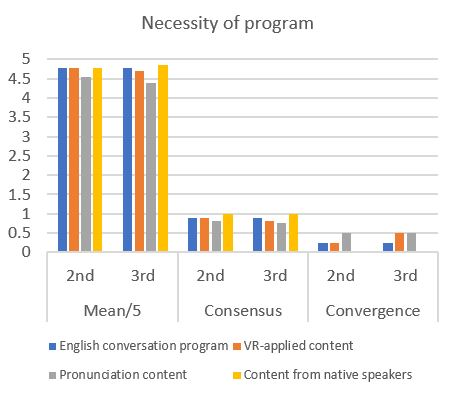

Supplement: S3 Fig — (TIFF) [file pone.0264850.s003.tiff]

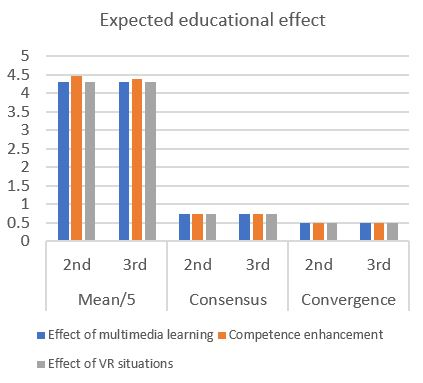

Supplement: S4 Fig — (TIFF) [file pone.0264850.s004.tiff]

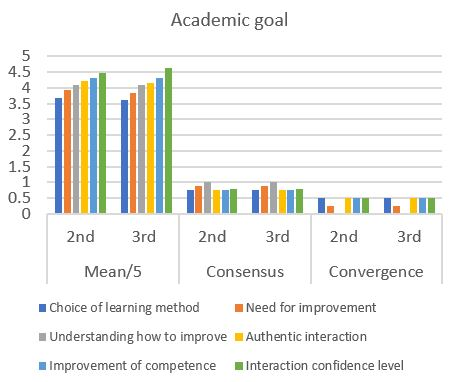

Supplement: S5 Fig — (TIFF) [file pone.0264850.s005.tiff]

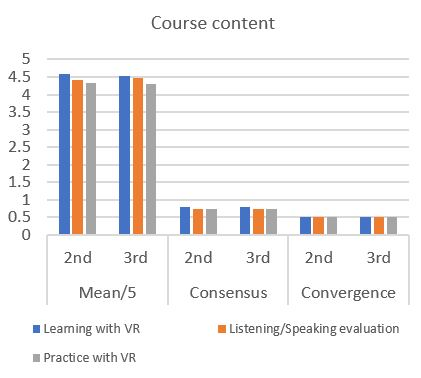

Supplement: S6 Fig — (TIFF) [file pone.0264850.s006.tiff]

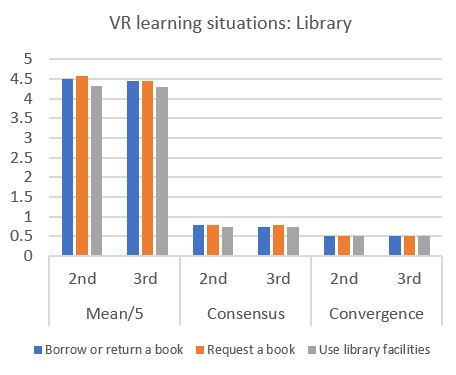

Supplement: S7 Fig — (TIFF) [file pone.0264850.s007.tiff]

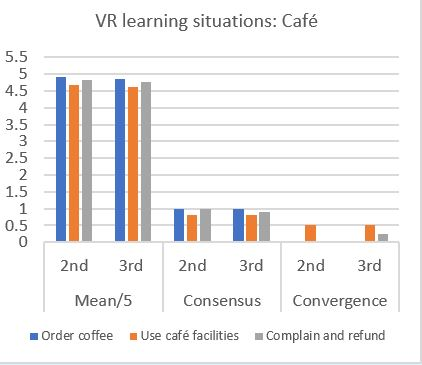

Supplement: S8 Fig — (TIFF) [file pone.0264850.s008.tiff]

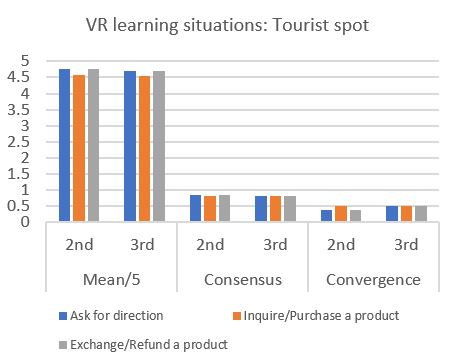

Supplement: S9 Fig — (TIFF) [file pone.0264850.s009.tiff]

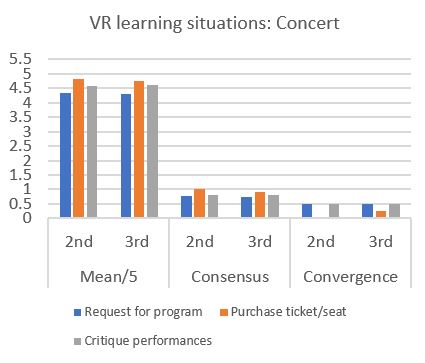

Supplement: S10 Fig — (TIFF) [file pone.0264850.s010.tiff]

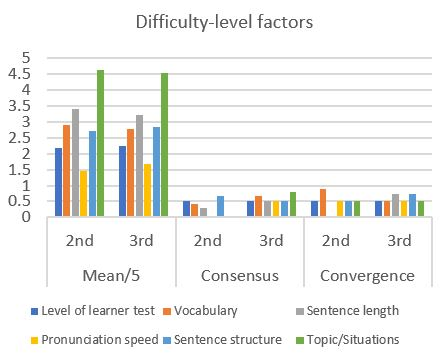

Supplement: S11 Fig — (TIFF) [file pone.0264850.s011.tiff]

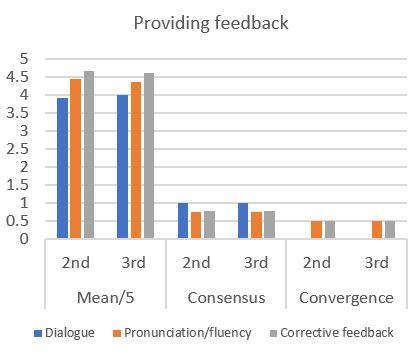

Supplement: S12 Fig — (TIFF) [file pone.0264850.s012.tiff]

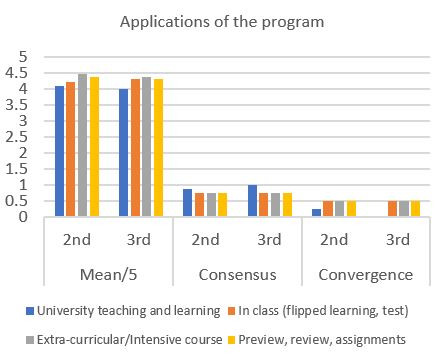

Supplement: S13 Fig — (TIFF) [file pone.0264850.s013.tiff]
